# Evaluation of Anthropogenic Substrate Variability Based on Non-Destructive Testing of Ground Anchors

**DOI:** 10.3390/ma14185131

**Published:** 2021-09-07

**Authors:** Marek Wyjadłowski, Janusz V. Kozubal, Zofia Zięba, Dmitri Steshenko, Dariusz Krupowies

**Affiliations:** 1 Faculty of Civil Engineering, Wrocław University of Science and Technology, 50-370 Wroclaw, Poland; janusz.kozubal@pwr.edu.pl; 2 Department of Civil Engineering, Wrocław University of Environmental and Life Sciences, 50-375 Wroclaw, Poland; zofia.zieba@upwr.edu.pl; 3 Transport and Mechanical Engineering Institute, North Caucasus Federal University, 355029 Stavropol, Russia; dmitristeshenko@mail.ru; 4 Copernicus Airport Wrocław, 54-530 Wroclaw, Poland; d.krupowies@wrolot.com.pl

**Keywords:** ground anchors, load test, creep phenomena, anthropogenic soil parameters

## Abstract

The purpose of this paper is to describe the variability of soil rheological properties based on research carried out using load tests of ground anchors under complex geotechnical conditions. The heterogeneity of soil should always be considered when designing geotechnical constructions. In the present case, the earthwork created at the Warsaw Slope revealed an embankment of anthropogenic origin, located in a geologically and geomorphologically complex area of the Vistula valley slope. Excavation protection was anchored mainly in soils of anthropogenic origin. When the acceptance tests of the ground anchor were completed, the subsoil randomness was confirmed using nondirect, geostatistical methods. A standard solid rheological model with nonlinear fitting to the data was used. This model was established to describe the creeping activity of the ground anchor more accurately. The characteristics of man-made embankments were described using the parameters obtained with the rheological model of the subsoil.

## 1. Introduction

In this paper, the problem of recognizing substrate variability is investigated using a nondirect method. This task is often associated with difficult access to the adjacent subsoil, conservation or preservation of buildings. There are some relevant methods for these tests is the measurement of displacement or strain. One of the common applications of geodetic methods in relation to geotechnical structures is the measurement of the indirect determination of the material properties of a substrate. Nondestructive testing of geotechnical structures provides data on the structure–soil interaction. The scope of displacement and deformation of soil–structure systems [1] used for slow-variable phenomena. Deformation analysis based on multi-temporal terrestrial laser scanning (TLS) surveys has been applied for many years to investigate commercial and academic problems [2]. Close-range photogrammetry with image processing can be used to accurately measure ground and structure deformation [3]. Geodetic methods have found direct application in the design process (observational methods) [4], as well as in structure monitoring and maintenance control [5]. It is difficult to use conventional instruments to measure the shear stress (deformation) along geotechnical structures, such as pile foundations, soil nails and retaining walls, due to their inherent disadvantages, such as large size and electromagnetic interference. Difficult access and problems with securing wires and connections during construction also contribute to the complexity of this process. To overcome these obstacles, fiber Bragg grating (FBG) sensors are recommended for measuring the shear stress along a pile anchor in pull-out tests. The different types of fiber-optic sensing technology include FBG, low-coherence interferometry (LCI), optical time domain reflectometry (ODTR), Fabry–Perot interferometry (FPI) and Brillouin scattering. In [6], a series of pull-out tests were conducted on a model pile in soil with different initial water contents and different pile surface roughnesses, ranging from smooth to rough, to study their influences on pile skin friction. Changes in axial strain and skin friction were measured by FBG sensors using a laboratory test procedure. In this paper, the concept of an analytical diagnostic tool for soil anchors is presented. Anthropogenic ground, which is often difficult to identify in practice, with numerous overlaps and discontinuities, was treated together with anchorage material. Information about the variability of the characteristics of the homogenized medium was obtained. In addition, the authors noticed many possibilities of applying the diagnostic method to the inspection of historical objects. In the presented work, it is the object and its scale that drive the search for new methods to describe variability. Scientific information on both the spatial variability and distribution of anthropogenic soil properties is necessary to model soil–structure systems and to make decisions regarding the need for modifications, strengthening or replacement [7]. Geotechnical engineering has a considerable arsenal of methods for parameter modification. However, little is known about the spatial distribution and variability of soil properties on the scale of the study site, and historical data queries introduce even more uncertainty about quality. This information is confirmed by geological and geotechnical reconnaissance. The purpose of this study is to investigate the spatial relationships and variability of bedrock properties using geostatistical methods and by means of nondestructive measurements of rheological characteristics.

For decades, the area of the capital city of Warsaw has undergone intensive urbanization processes, including a constant influx of people, the development of processing industry, the extraction of mineral aggregate and damage from military conflicts, as well as subsequent reconstruction. These activities caused major changes in the natural environment, especially in the subsoil. Anthropogenic changes in the environment with respect to geological structure and terrain relief, climate, hydrographic conditions, soils and flora have considerable influence on civil structures. Areas with anthropogenic soils had already been marked in the geological documentation of Warsaw in 1937 [8]. Urbanization processes have been occurring since the establishment of the city at the turn of the 13th and 14th centuries, peaking in the 19th century and in the 1970s of the 20th century. 

**Figure 1:**
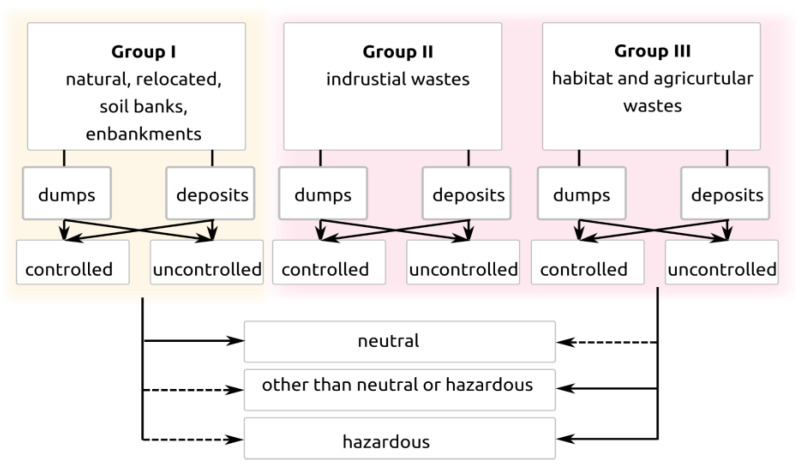
The division of anthropogenic soils according to standard [6].

The contemporary environment is not free from anthropogenic transformations, and the earthwork in question is an example of this. Anthropogenic changes also take place on the surface of the terrain, where they have an impact on civil structures. Many geologists [9], construction engineers [10,11] and geographers [12] have examined anthropogenic soils in urbanized areas, as well as outside of them. The high interest in this matter is due to the highly diverse properties of soils that make up subsoils with altered characteristics owing to human activity. These soils are a lithological foundation, especially in urbanized areas, and a subsoil for engineering constructions. Their presence also indicates the transformation of the terrain relief. Morphodynamic processes, which are hazardous to the environment, occur in areas with anthropogenic soils and require the thorough design of geotechnical structures. The characteristics of anthropogenic embankments indicate their diverse composition in terms of both vertical and horizontal distribution. Some of these embankments are natural soils of various origins and compositions (obtained from different sites and deposited), and some are products of human activity. During the long process of city development, the material itself and the means of its accumulation underwent many changes. It was influenced, among other events, by fires, floods and damage that occurred over the centuries. The task of extracting anthropogenic soils from backgrounds of different origins is often complex. The relative proportions of anthropogenic material and the soils of natural origin reveal anisotropy in both the vertical and horizontal directions. The upper part of the anthropogenic layer usually has a higher level of heterogeneity of mechanical characteristics in the plan and contains more material with higher average grain size distribution. The classification of soils is specified in the PN-EN ISO 14688-1 standard [13]. A schematic division of the soils according to this standard is shown in Figure 1 below.

Only the following types of soil can be considered for the construction subsoil: anthropogenic soils in tiers I and II, present in the form of post-mining and post-energy sites [14], the construction debris of the re-cultivated excavation sites in tier III, and macrolevel areas. It is recommended to increase the development of foundations with low load per unit area, with the type of foundation adjusted to the heterogeneous subsoil, namely, a strip foundation with enhanced reinforcement, grillage or slab foundation. Anthropogenic soils in tiers I and II have become increasingly popular as a construction material for engineering objects, such as railway and road embankments, dams with small damming, embankments of wet landfills or levees. These soils are used in technical recultivation, for example, to fill troughs above underground coal and sulfur excavation sites and to fill the excavations themselves. They are also used as an insulation layer or an ingredient of mineral insulation layers and as an ingredient of soil compositions to increase their endurance and physical characteristics. When it comes to soils in tier I, two types of embankments should be distinguished: a controlled and uncontrolled one, with the former being an embankment formed according to a particular technology, in which its geometry, density and the assumed forming techniques can be controlled. 

Anthropogenic soils come in various forms, depending on the technology of their storage (dumping) and usage. At present, dumping (external and internal) is used to create landfills [15,16], heaps and earthwork structures [17,18,19] (e.g., road embankments, levees). Anthropogenic soils, as human-occupied layers, have also shaped the surface and subsoil in cities. Geological engineering research on these soils should be carried out in cooperation with archaeologists. Traces of human activity are often the criterion that shows the anthropogenic origin of geological layers. Due to the many possibilities of anthropogenic soil usage, the research aims and scopes are extremely diverse [20,21]. When it comes to engineering constructions, as with natural or native soils, it is crucial to investigate compaction parameters, as well as the choice of equipment and compaction technology [22], but it is also important to take into account the possible geochemical transitions of built-in soils and the degree of their aggressiveness towards concrete and steel constructions.

Soil transformation in processes I and II has a substantial influence on the engineering solutions used during the transitions and renovations of buildings. This requires a specific understanding of geodiversity, which synthesizes information on the abiotic layers and reveals the spatial interactions and mutual connections [23,24].

The complex composition of the Masovian Lowland, which, as a whole, contains traces of glacier activity from the Riss glaciation, as well as signs of accumulative and erosive activity of river and glacial waters during the interglacial periods [25], makes geotechnical conditions more complicated. Previous geological documentation presents the consequences of processes that have been transpiring for millions of years, and only in the last few centuries have there been substantial changes to the structure, composition and morphology of this terrain. During the construction and the many years of use of the Warsaw Citadel, different substances were introduced to the environment, including ammunition, waste, food and construction materials [26]. With the development of fortification techniques and the changes taking place in the international arena, the fortress had been expanded by the erection of forts and external positions near the end of the nineteenth century, which required a huge scope of earthwork. It must be highlighted that anthropogenic interference is rather shallow, and on the profile scale, it concerns the surface layer only. Ground fortifications are still visible today and are a historic monument [27]. The study of the development of earthwork methods has led us to the conclusion that contemporary knowledge of the parameters and mechanics of soils is slowly changing the tradition of raising earth constructions using empirical experience and corrections during the time of use. When designing ground anchors and the soldier pile wall, both the geodiversity and the anthropogenic origin of soils should be taken into account [28]. The influence of anthropogenic soils on the design of geotechnical constructions was shown in [29], among other studies. Studies on deformations in retaining structures and retrospective analyses have shown that anthropogenic soils that had initially been classified as low bearing have presented average durability parameters.

The presence of cultural layers is highly visible when performing deep excavations that reveal the spatial heterogeneity of the ground and its interactions with contemporary constructions.

## 2. Research Object

The geotechnical material under investigation originates from the historical embankments of a citadel located in Warsaw, the capital city of Poland, located in the Mazovian Lowland. The trough is filled with sediments from the Cretaceous, Tertiary and Quaternary Periods and shaped by geomorphological processes that took place in the Quaternary. The main geomorphological units within the city of Warsaw are the Warsaw Plain, the Wołomin Plain and the Central Vistula Valley. The Warsaw and Wołomin Plains were created by glaciers in the Riss glaciation period and by the accumulative and erosive activity of river and glacier waters in the interglacial periods. The predominating materials in the area of the Warsaw and Wołomin Plains are the Pleistocene glacial tills (from sandy clays through clayey sands). The highest peak of the plain is about 115 m above sea level and is located in the city center. In the East, the plain is divided by the Vistula river, where the Warsaw Slope’s cliff is located [30,31]. The view of the Warsaw Slope with the city panorama is characteristic of the city. The slope divides the city into north/south sides. Recreational areas, parks and palaces that survived WWII are located nearby. Currently, the Vistula valley is created by the riverbed and the following terraces: floodplain, fluvial, dune and lacustrine. Most of the right-bank part of Warsaw is situated on the terraces along the Vistula river. These terraces are made of alluvial sediments, such as gravels, sands, river sediments, fluvisols and organic sediments. Dune and eolian sands are present in the fluvial terraces and make vast areas of sorted sands and dunes. The citadel was raised in the left-bank part of Warsaw, directly by the river bed, in the area of the Warsaw Slope and the Warsaw Plain terraces. In 1830, the November Uprising against Russian domination broke out [32]. Warsaw became the most important and long-lasting point of resistance for the uprising. As a result, the construction of a stronghold that could be a resistance point for the Russian army guarding the capital city of the Kingdom of Poland became a necessity. It had an entirely non-metropolitan character and function; however, due to modifications to its military functions and the endurance of its technical substance, it has managed to survive to this day [33].

The Warsaw Citadel was raised in the Żoliborz district, which was located in the 16th-century Fawora manor farm and the Piarists Monastery from the 17th century [34].

Additionally, during archaeological work before the museum construction, a former Protestant cemetery was discovered [35].

**Figure 2:**
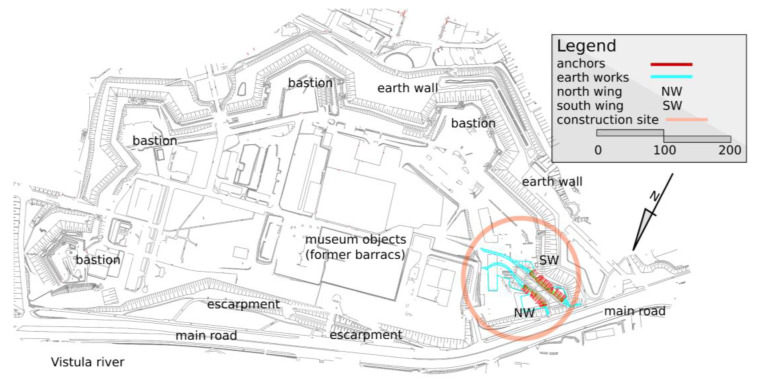
The detailed plan of Warsaw Citadel based on the design basis map (in Poland) at a scale of 1:500. The circle contains a newly designed entry into the backyard of the object.

The construction of the Citadel in place of the Royal Guard barracks was initiated by Tzar Nicholas I in 1832. The Citadel has the shape of a semicircle, whose chord is based on the river (Figure 2). The whole stronghold is surrounded by a scarp with a dry moat and counterscarp. There are three bastions and two half-bastions. The arms of each of them included a single-level cannoneer position. The solutions used in the Citadel were based on schemes popular in the 19th century [36,37]. There was neither a long-range defense system nor protection from the artillery, no ammunition bunker and no defensive barracks. The ceilings had no earthen ramparts, with only a thin roof covered with ceramic ones for larger areas. The Citadel was not meant to protect against the regular army, but only against insurgents with no artillery whom the Tzar expected to come from the conquered Warsaw. It was designed as a display of the power of the Tzar and to provide a vast and capacious space. On the first day of the January Uprising, the garrison had 16,000 soldiers. A crucial element of the Citadel was its artillery, whose aim was to keep the center of Warsaw under control: this threat worked well during the January Uprising, as the temporary government decided not to provoke a rebellion in the city [38].

**Figure 3:**
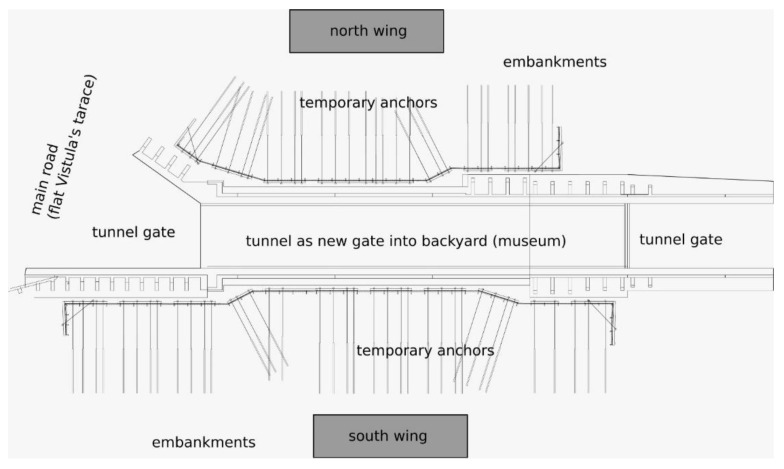
The designed entry to the Citadel with the protection of the excavation in the anthropogenic soils (anchored soldier pile wall).

**Figure 4:**
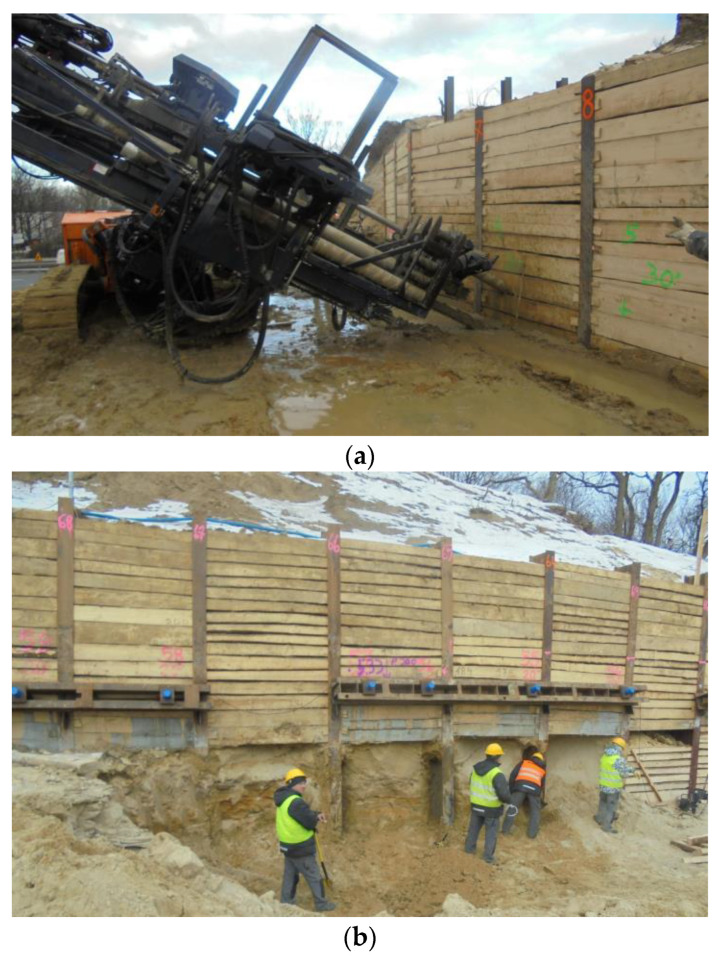
The pictures show (**a**) soldier pile wall and installation of the ground anchors, (**b**) mounted heads of the anchors.

Architects of the WCXA studio are transforming the Citadel into a tourist attraction and a memorial. The new museums that will be located there will change the defensive character of the land development. The walls and ramparts must be open for vehicle and pedestrian passage (Figure 2 and Figure 3). Geotechnical works, as a stage of this project, are shown in Figure 4.

According to a design concept selected in an architectural contest in 2010, the following parts of the Warsaw Citadel will be used for museum purposes: 

The Caponier, which will be the head office of the Katyń Museum, with a permanent exhibition [39]; the Shoulder Battery; and the Nowomiejska Gate (the main entrance to the museum and the open-air area). In 2022, the Museum of the Polish Army will be fully relocated to the Warsaw Citadel. The new facilities will generate significant visitor traffic, especially car drivers, hence the concept of opening up the site and building a new tunnel through the earth embankment. 

## 3. Geotechnical Site Investigation

**Figure 5:**
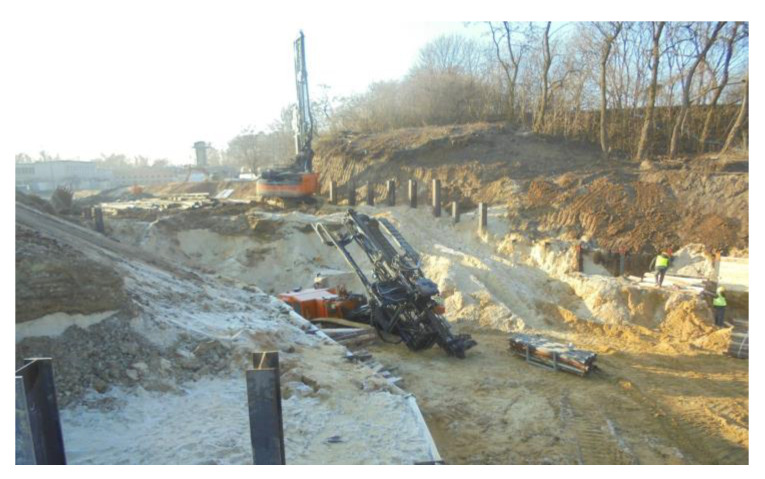
Passage of the communication corridor through the anthropogenic embankments: construction of the soldier pile wall. Clearly visible uncovered substrate layers of different colors.

The geotechnical conditions in the substrate and the quality of the material become important in the construction and use of massive structures, including fortifications, in particular embankments and the protection of deep excavations. The variable layering visible during the earthworks (Figure 5) is a confirmation of the former geotechnical investigation.

The area of Warsaw Slope terraces includes regions that have active erosive processes and creeping movements, as well as places where there are springs, wetlands areas and depressions of suffosion origin. These factors adversely affect the foundations of buildings and engineering structures, in particular:

- Landsliding phenomena;
- The occurrence of an anthropogenic soil layer in the substrate;
- The occurrence of a layer of low-bearing soils in the substrate;
- An underground water table with a shallow level.

Geotechnical investigations of the substrate in the area of the designed cutout included the geotechnical drilling hole and cone penetration tests (CPT) [40]. As a result of anthropo-pressure, which especially intensified during the Citadel’s erection, the primary terrain was leveled and additionally reinforced by building up a layer of anthropogenic embankments. The sediments lithologically comprise the following materials: clayey sands, sandy clays and silty clay with stiff-consistency gravels and cobbles. All of the above-mentioned sediments are of Quaternary origin. Older subsoil, i.e., from the Tertiary and Pliocene periods, was found at the bottom of the scarp.

**Figure 6:**
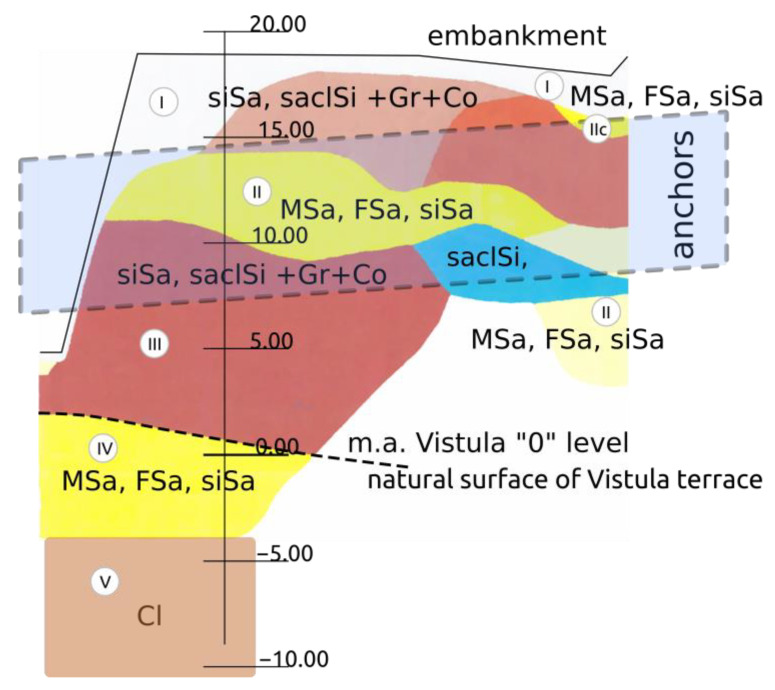
Cross-section along the road support (with courtesy of Geoteko data [40]).

For design purposes, the following basic geotechnical layers have been established (Figure 6):

- Layer I: from the top, a layer of humus and anthropogenic embankments up to 5.0 m thick. The material of this layer is heterogeneous and contains a significant share of brick rubble.
- Layer II: fluvioglacial sands of the Wartanian Glaciation, medium sands with density index *I_D_* = 0.4–0.80.
- Layer III: unconsolidated tills of the Wartanian Glaciation, formed as clayey sands and sandy clays with gravels and cobbles with a stiff consistency and plasticity index *I_L_* = 0.1–0.2.
- Layer IV: glaciolacustrine deposits of the Wartanian Glaciation formed as silts, sandy silts, silty clays and clays with a stiff consistency.
- Layer V: consolidated cohesive glacial deposits of the Odranian Glaciation consisting of sandy clays and clayey sands with gravels and cobbles with a very stiff consistency.

For the design task under consideration, the layering between the two drilled test holes was adopted. Reconnaissance was carried out to confirm the adequate capacity of the soil anchors and to ensure their durability for a period of two years. As mentioned above, the embankments are made of local soils found close to the fortifications. The substrate classified as original can also be anthropogenic. The current layer layout is revealed only at the time of excavation. The carefully observed walls of the excavation make it possible to view the true arrangement of geological layers, often different from the one drawn on the basis of research drills. The attempt to adopt horizontal layer boundaries is visible in most geological engineering cross-sections included in the documentation. These basic assumptions work well in the case of a simple and complex geological structure, but in the case of a complicated structure, due to the glaciotectonic origin of ground displacements, they are not always correct. An example is the geological structure presented in the cross-sections generated for the needs of the renovation works under consideration. 

**Figure 7:**
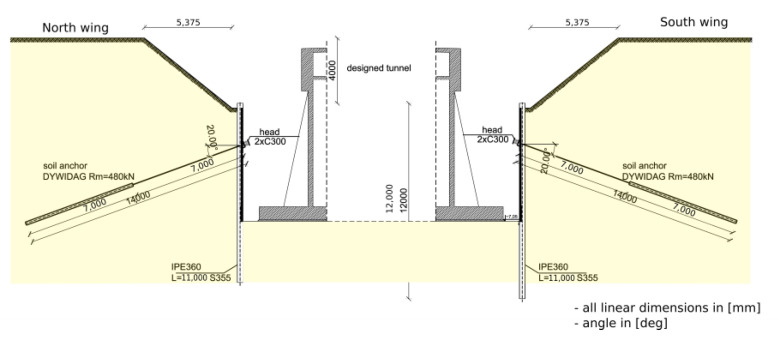
Details of excavation protection: design phase, with location of anchors in typical cross-section (linear dimensions are in [mm], angles in [deg]).

The described adaptation requires a cross-cut in the embankment and protection of the excavation in the embankment. The first stage of implementation of the entrance to the designed museum object requires the demolition of approximately 54 m of the Carnot wall. The excavation under investigation was carried out in an uncontrolled anthropogenic substrate and in non-cohesive and cohesive subsoil. The task requires the identification of a substrate made of anthropogenic soils in which excavation and estimation of geotechnical parameters will be carried out. After the Carnot wall had been demolished and the soldier pile wall was completed, the work was in progress to create the excavation. The further stages of the structure are presented in Figure 5 and Figure 7.

A soldier pile wall is predominantly a temporary structure, and it usually stays in place after the excavation work is completed. The lifetime of the structure is limited, and no changes in geotechnical soil parameters during use are predicted. Prestressed anchors were introduced in one row at an interval of 2.5 m. For anchoring of the soldier pile wall, Dywidag Strand Anchors (Dywidag Prestressing Steel, 140 mm^2^ Number 3) were used: anchors with a 7.0 m long bond length and 480 kN. Fixed anchors are located in different soil layers with variable physical properties. The location of 7.0 m long, slanting fixed anchors is shown between the marked lines in the picture (Figure 6). The fixed anchors are positioned in the zone of occurrence of the Wartanian Glaciation’s sediments, represented by fluvioglacial and glaciolacustrine sands (fine sands, silty sands and medium sands), glacial tills (clayey sands, sandy clays with gravels, cobbles and boulders) and locally lacustrine soils (silt, silty clay and clay). The total thickness of these sediments ranges from 5.0 to more than 10.0 m. The anchor capacity was predicted and designed as a deterministic value based on empirical formulation. The final verification of anchorages was subsequently performed using creep tests after pre-compression of anchors by a static pull-out load test.

## 4. Anchor Test Description

### The Principles of Design and Testing

**Figure 8:**
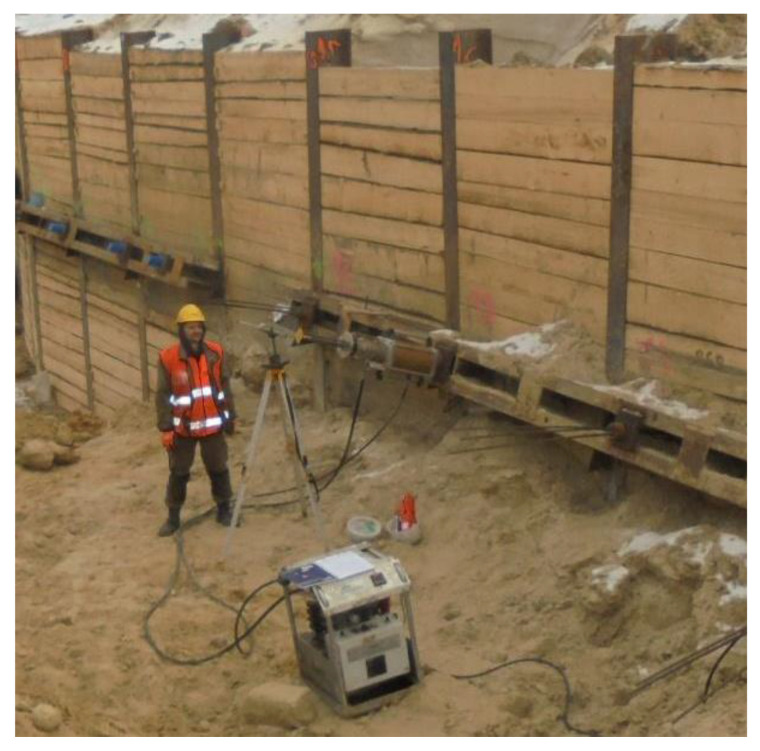
Details of excavation protection: load test of ground anchors.

As part of site investigations, excavations, ground anchor testing and other engineering works, boreholes are crucial for understanding Warsaw Citadel’s geodiversity. The designers used anchors as temporary supports for soldier pile walls (Figure 5). During construction, it was possible to look closer at the substrate and uncover some mechanical dependencies [41,42]. The standard [43] provides the general definition: an anchor is a structure capable of carrying tensile loads through a free length of a tendon onto a support layer. On the other hand, for an injection anchor, it is specified that an anchor with a bolt solidified by the injection of resin, cement grout or other material transfers the tensile force to the ground. The standard [44] applies to temporary or permanent soil anchors that are ground-bonded by injection, compressed and tested according to the strictest safety measure described. The field test of the anchor head is shown in Figure 8. The measuring set consists of a dial indicator sensor and an actuator (conventional hollow-piston cylinder).

Ground anchors mainly carry pulling forces, sometimes slightly transverse, and do not transfer compressive forces [45]. The main areas of their application include the stabilization of an excavation and other support structures. This stretch does not make the surrounding soil stronger. Typical ranges of anchor load capacity vary from 500 kN to 2.0 MN. The main document referenced for the testing procedure in this study is the standard [46]. It presents a complete range of design calculations and implementation procedures for both ultimate and serviceability limit states. It concerns only the design and testing of anchors with free length. However, at present, there is no effective method for monitoring the anchors. The three coordinated standards [43,44,46] assessing the design, execution and testing of grouted anchors, respectively, in the EU, establish three types of tests: investigation tests, suitability tests and acceptance tests. The standard [46] includes 3 test methods: TM1, TM2 and TM3. All of these tests are designed to evaluate the global performance of the anchors by taking external measurements (e.g., applied force, relaxation and creep). In particular, the investigation test is a load test aimed at evaluating the ultimate geotechnical resistance of the anchor and its behavior at working loads.

**Figure 9:**
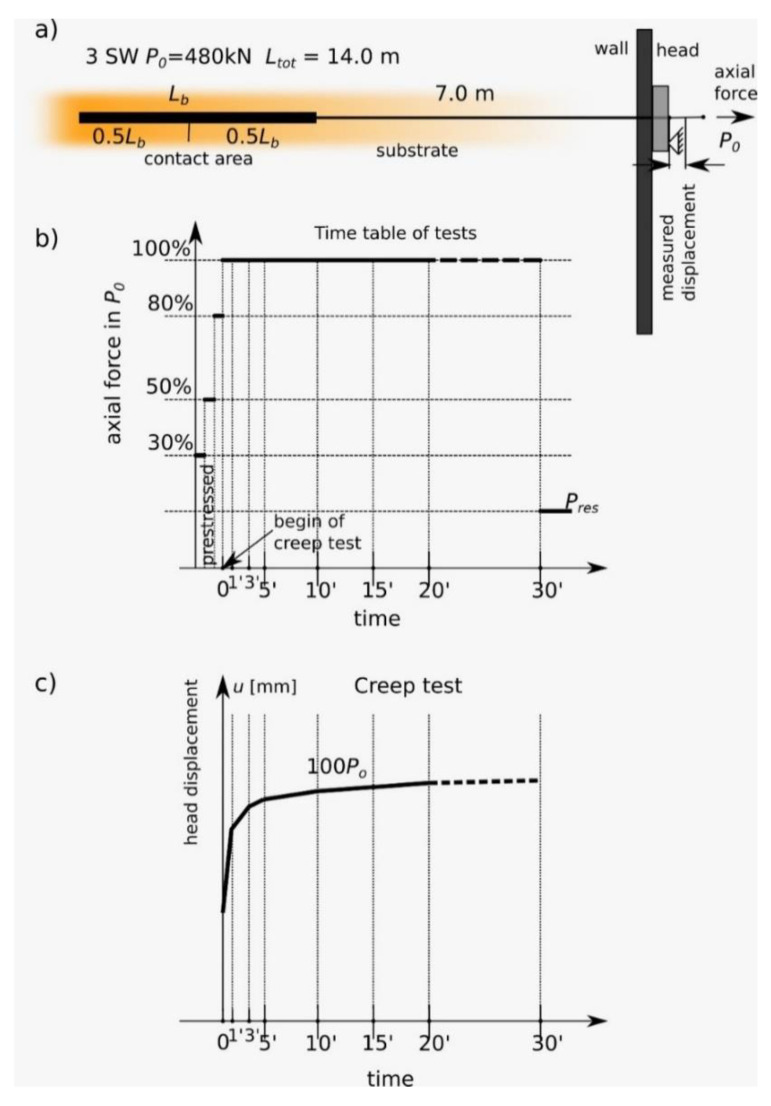
Example of a graphic anchor creep test report: (**a**) profile of 3SW-type anchors, which were used in construction and tested; (**b**) schedule of load–time procedure in experiments; (**c**) typical creep result chart.

This article discusses an acceptance test according to the TM3 model (Figure 9b). The procedure must be carried out on all anchors. The anchor is loaded in incremental steps from a datum to the maximum load (here, the design capacity of the anchor). The incremental steps were 10%, 30%, 50%, 80% and 100% of the design capacity. The displacement of the anchor points was measured under a maintained load at each loading step. The measurements summarize the anchor head displacement versus anchor load at the beginning and end of each load step.

After reaching the test tension, the creep rate α is determined by:

(1) $$\alpha =\frac{\left(S_{2}-S_{1}\right)}{\log\left(\frac{t_{2}}{t_{1}}\right)}$$

where *S*_2_ and *S*_1_ are creep displacement at times *t*_2_ and *t*_1_, respectively.

The anchors were installed using the Hütte HBR 609-3 set. This is a hydraulic drilling rig for micropiles and ground anchors, foundation construction, soil investigation and slope stabilization. The most important parameters are engine power 209 kW, extraction force 100 kN, crowd force 100 kN and working extraction speed 17.5 m/min. The diameter of the casing used was 133 mm. The device performs the entire technological process of creating the anchor quickly and efficiently. The anchors were drilled with a gimlet bit in pipe casing with water flush. Upon making a borehole, grout injection was carried out and then the anchor bearing element—a set of wires—was installed with double corrosion protection (DCP) to ensure a prolonged lifespan; the element was equipped with spacers, injection hoses and separator(s). Primary pressure grouting was applied through the casing every 2 m during its removal, and then the pressure applied was 1 MPa. After approximately 24 h, all anchors were grouted by tube-a-manchette with a pressure of 6–7 N/mm^2^. During primary and secondary pressure grouting, the grout used had w/c = 0.50 and was produced from CEMII 32.5R. Finally, 7 days later, the anchors were tested, compressed and stabilized with anchorage (Figure 9a). 

System creep tests were performed after pre-compression of anchors.

## 5. Modeling Methodology for Time-Dependent Process

The genetic and geomorphological diversity of the subsoil material not only affects the physical characteristics of the substrate in which the fixed anchor tests are performed but also presents difficulties in describing its rheological characteristics [47]. There are many methods of creep testing for soils and soft rocks: these are methods for loading large-scale cylindrical loads [48]. They require a long time with a special apparatus and a constant climate (controlled pressure, temperature and humidity). Research procedures are available in [49]. As a different approach, forced testing after destruction on the direct surface of slip is possible in a modified rotary shear apparatus [50]. In the case of anchor tests lowered in the ground at the construction site, an attractive option is the in situ test presented in the article. The authors used the method of direct measurement of these properties based on creep tests [46]. The standard linear solid model of viscoelastic soil [51,52] was used. The Hook element (first spring $\gamma_{1}$) was connected in series with the Kevin-Voigt element (second spring $\gamma_{2}$ and first damper $\theta_{2}$). The strains of the elementary model components (Hook and Kevin-Voigt) were summarized as a one-dimensional process, along the anchor tendon, to the total value. The calculation was conducted for a constant load value in the anchor head, where stress is σ = P_0_/*A*, with *A* as the cross-sectional area of the anchor tendon and *P*_0_ as the axial force. According to the Code [46], the total time of the experiment was 30 minutes (Figure 9c), and the lack of long-term creep results was addressed by the assumption that σ is constant, where σ represents compressive stress. The boundary task has strain ε(t) equal to $\varepsilon_{0}$ at time *t*_0_ = 0, and it allows us to determine the constant of elasticity of the first spring *γ*_1_ (Hook’s element) as $\gamma_{1}=\sigma /\varepsilon \left(0\right)$.

## 6. Results

Measured displacement *u* [mm] in this study was divided by the fixed anchor length *L_tot_* for calculating the anchor strain. This assumption was made because the anchor–tendon–soil system was analyzed. The Kevin-Voigt part has two sub-elements: liquid and solid body types. Their stress values are summarized with the output values. Simultaneously, the strains of these two submodels are equal. The first-order differential equation was obtained as:

(2) $$\left(\varepsilon -\frac{\sigma }{\gamma_{1}}\right)\gamma_{2}+\dot{\varepsilon }\;\theta_{2}=\sigma .$$

At the final time of the experiment $t_{l}$, it was assumed that the second damper had disappeared $\dot{\varepsilon }\left(t_{l}\right)=0$, which allows the second spring coefficient $\gamma_{2}$ to be deduced from (2):

(3) $$\gamma_{2}=\frac{\sigma }{\varepsilon \left(t_{l}\right)-\varepsilon \left(0\right)},$$

which allows the solution to be obtained with one unknown parameter $\theta_{2}$:

(4) $$\varepsilon \left(t\right)=\varepsilon \left(0\right)+\frac{\sigma }{\gamma_{2}}\left(1-e^{-\frac{t\gamma_{2}}{\theta_{2}}}\right).$$

**Figure 10:**
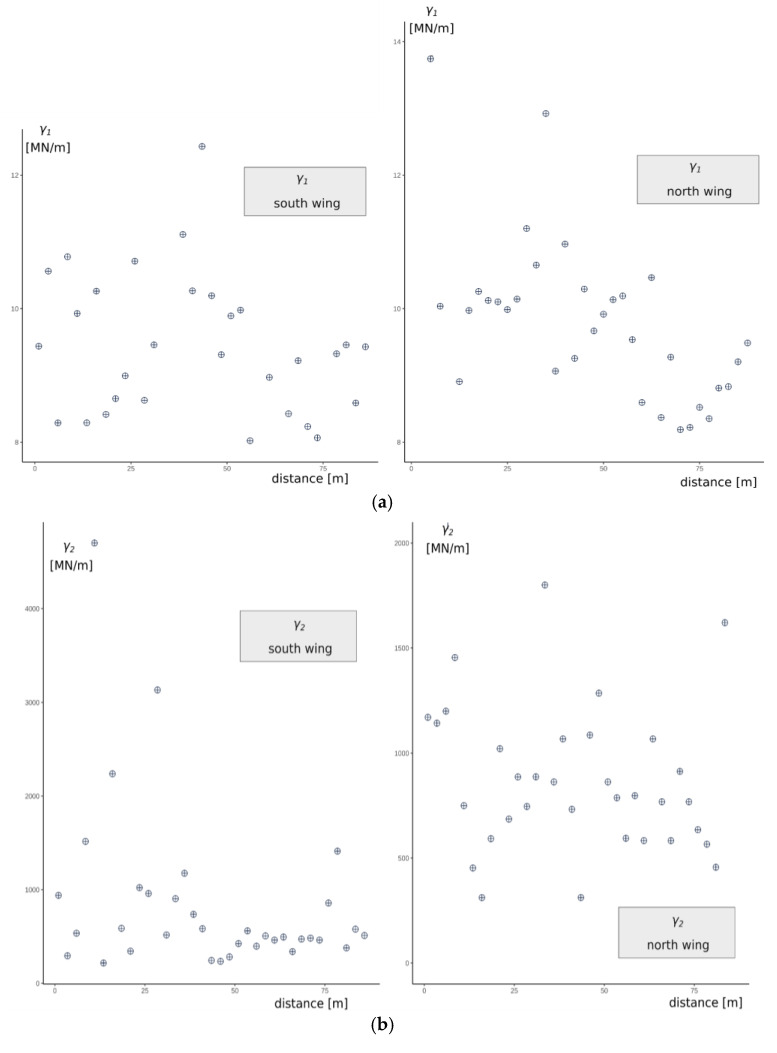

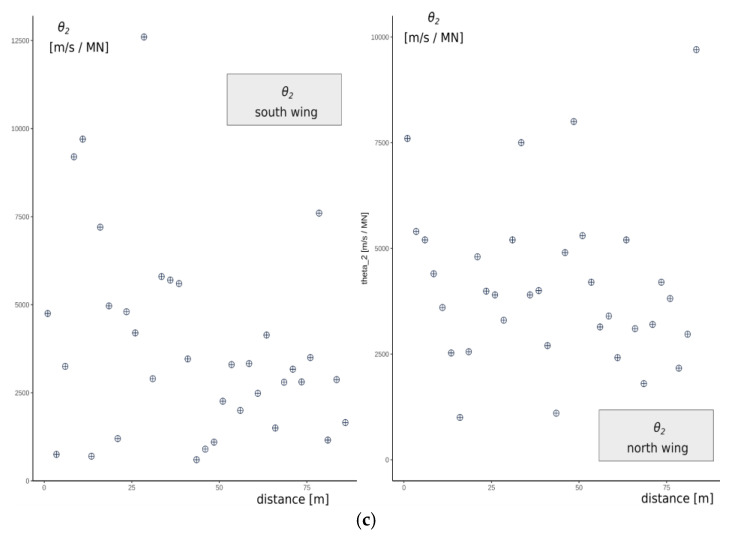
Values of (**a**) $\gamma_{1}$, (**b**) $\gamma_{2}$, (**c**) $\theta_{2}$ plotted against anchor position for south and north wings.

To test the spatial variability of the model parameters, semivariograms were used and are presented graphically in Figure 10 and by direct statistical parameters in Table 1. The minimum distance between the testing points is compatible with the anchor spacing and is equal to 2.5 m. The semivariogram data were taken along the wall of the south and north wings of the excavation. The two sides constitute the cross-section of the embankment. The shape of the semivariograms does not allow for building a model for the empirical variability of the tested characteristics along the cross-section of the excavation. For both sides under consideration, the randomness of the model parameter value is quite high, and along the walls of the excavation, it can be regarded as white noise, with no functional correlation between the variance and the distance. This confirms the previous assumptions that the soils creating the embankment are highly heterogeneous, which also makes the distinction of homogeneous layers in the embankment difficult.

The correlation between model parameters was examined, and the results are presented in Table 2. The model parameters $\gamma_{2},\theta_{2}$ have a significant positive correlation. The statistically significant dependence of parameters describing the viscous part in the model indicates a similar material characteristic for time-dependent strains.

**Figure 11:**
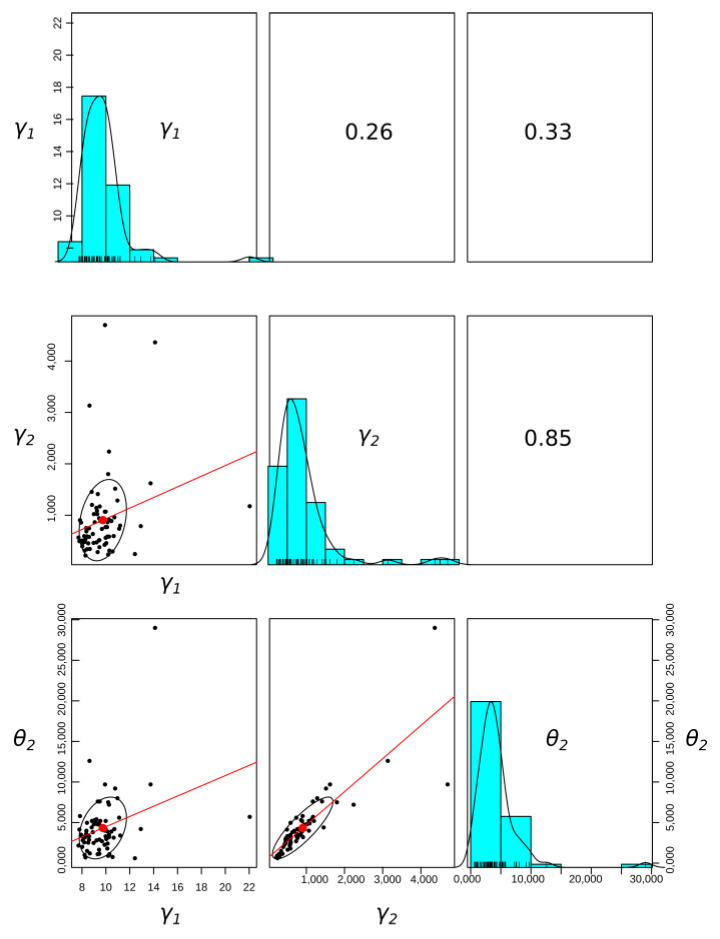
Graphical representation of Pearson covariance matrix with histograms. Fitting of linear relationships between parameters was performed for parameter values from both wings. The position of the graph in the figure is indicated by (row,column): (1,1) histogram of $\gamma_{1}$, (2,2) histogram of $\gamma_{2}$, (3,3) histogram of $\theta_{2}$, (1,2) correlation coefficient between $\gamma_{1}$ and $\gamma_{2}$, (1,3) correlation coefficient between $\gamma_{1}$ and $\theta_{2}$, (2,3) correlation coefficient between $\gamma_{2}$ and $\theta_{2}$, (2,1) plot of $\gamma_{1}$ vs. $\gamma_{2}$, (3,1) plot of $\gamma_{1}$ vs. $\theta_{2}$, (3,2) plot of $\gamma_{2}$ vs. $\theta_{2}$.

Basic statistical relationships between model parameters are presented graphically in Figure 11.

For the analysis of the variability of anchorage characteristics for the south and north wing sections, a geostatistical measure of variability was used to take into account the mutual position of the anchors. The semivariogram describes the degree of dependence of the feature (rheological and elastic parameters) as a function of distance in normalized Euclidean space ||*h*|| for isotropic phenomena, or as a function of distance and direction when anisotropy is assumed for phenomena in two or more dimensions. The semivariogram estimator has the form:

(5) $$2\gamma \left(h\right)=\frac{1}{N\left(h\right)}\underset{N\left(h\right)}{\sum }{\left(z\left(s_{i}\right)-z\left(s_{j}\right)\right)}^{2}$$

**Figure 12:**
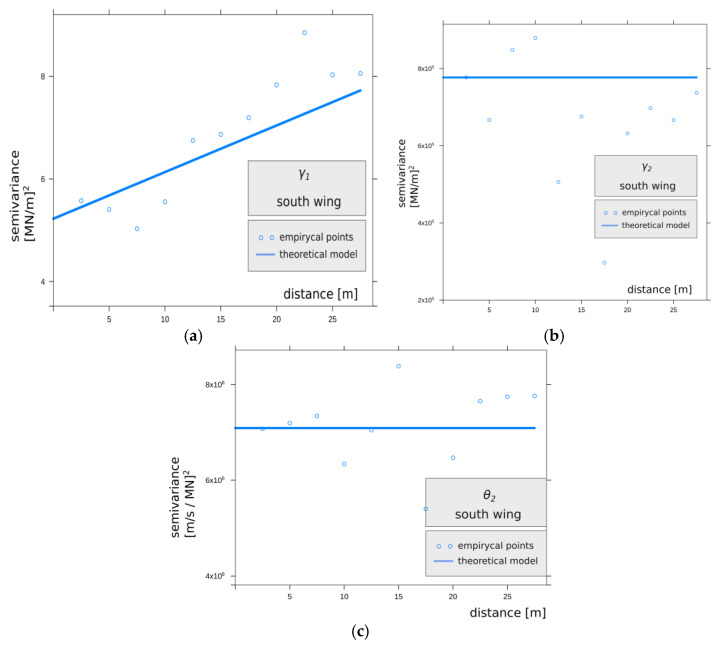
The graph shows points on the empirical semivariogram for the south wing. The line was fitted by the LSM of the theoretical model for the parameters: (**a**) $\gamma_{1}$, (**b**) $\gamma_{2}$, (**c**) $\theta_{2}$.

**Figure 13:**
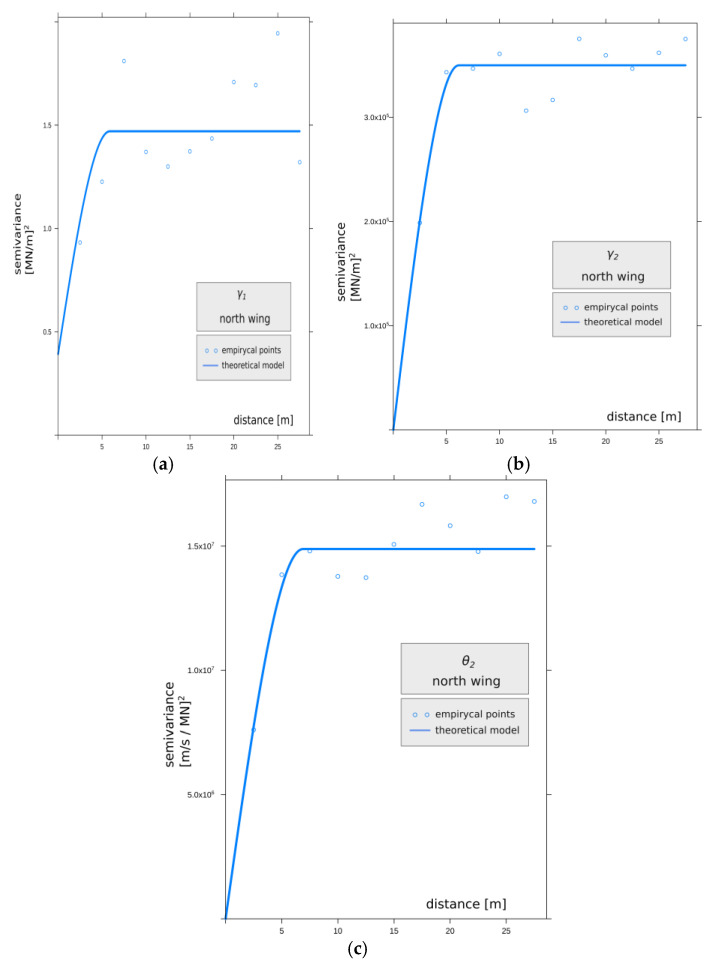
The graph shows points on the empirical semivariogram for the north wing. The line was fitted by the least square method (LSM) of the theoretical model for the parameters: (**a**) $\gamma_{1}$, (**b**) $\gamma_{2}$, (**c**) $\theta_{2}$.

In Equation (5), *N*(*h*) denotes the number of all pairs (*z*(*s_i_*) − *z(s_j_*))^2^ that are separated by distance ||*h*||; *z*(*s*) denotes the value of the feature at location *s*; *z* denotes the set of all discrete values of that feature; and *i*, *j* are natural numbers in the interval [1,*n*], where *n* is the number of measured points. For practical reasons [53] and the symmetry of the phenomenon with respect to zero spacing, a semivariogram, defined as half of the variogram *γ*(*h*), was used. The semivariogram is a measure of dissimilarity between points observed at a given location *z*(*s_i_*) and *z*(*s_j_*). Figure 12 shows points on the empirical semivariogram for the south wing, and Figure 13 shows points on the empirical semivariogram for the north wing. In both figures, the lines were fitted by the least square method (LSM).

The high value of the nugget effect in the semivariogram analyzed in Figure 12 and Table 3 and its linear theoretical model indicates significant randomness and diversity of the parameter $\gamma_{1}$ values for the south wing, while the character of the variation in this parameter for the north wing is presented differently, as shown in Figure 13 and Table 4. Here, the semivariogram is described by a spherical model, and a smaller value of the nugget effect can be observed, which means greater homogeneity of the material. Differences in the origin of the material and methods used to erect the embankments surrounding the Warsaw Citadel are evident on both sides associated with adjacent masses of anthropogenic embankments subjected to the analysis of the variability of elastic features of the rheological model of the subsoil. The spatial variability of the feature $\gamma_{2}$ in Figure 12b and Figure 13b and $\theta_{2}$ in Figure 12c and Figure 13c is supported by significant divergences between the analyzed semivariograms.

There was no significant correlation in the remaining combinations of parameter pairs.

A lack of correlation with the element responsible for immediate deformations $\gamma_{1}$ allows for the presumption of different mechanical processes affecting capillaries or grains and influencing the behavior of the material. The human influence on soil formation is much deeper and more intense than we can perceive. For this reason, it is imperative that anthropogenic processes are clearly recognized as a factor in soil formation.

In the present case, the design of the anchors and their execution took into account the presence of anthropogenic soils with a smaller range than was shown by the acceptance test results. In the performed tests, the load was not increased until reaching the ultimate or serviceability limit state of the anchor; therefore, it is not possible to indicate the mechanism of failure of the grout or soil here. Numerous test programs have been carried out for anchors in concrete and have shown that three different failure modes are considered, including concrete cone failure at the unloaded end without interfacial debonding, interfacial debonding plus concrete cone failure at the same height as the interfacial shear crack tip, and interfacial debonding plus concrete cone failure at the unloaded end. In theoretical studies, linear elastic fracture mechanics is generally used to calculate the cone failure load. A similar study was carried out for soil nails [43], the function of which is identical to that of anchors. The shear strength between the soil nails and the soil is an important parameter for the design of the soil nails. This parameter is governed by a number of factors, such as the stress condition, normal stress acting on the nail–soil properties (strength, particle size, soil dilatancy, degree of saturation, etc.) and nail–soil boundary conditions (surface area and roughness). Multiple nail-soil tests were performed in a pull-out box. Full-scale tests are presented in [47]. All anchors were tested according to [45]. The investigation test using the cyclic load test procedure was conducted, and after reaching the maximum level of the prestressing force in every cycle, the force was kept constant, and the displacement of the tendon was measured and recorded at specified times. In the tests [47], all boreholes for anchors were drilled vertically. Rotary drilling without casing was used to drill the boreholes; however, this is not typical for anchors that are drilled diagonally or horizontally.

A similar process of behavior under the loading and failure mode of tested anchors was observed on all tested members. After reaching the ultimate resistance (maximum value of the prestressing force), a sudden decrease in the prestressing force occurred. This was accompanied by a visible displacement of the grout body out of the ground.

The ultimate capacity of ground anchors is mostly determined by using various empirical and semi-empirical methods [54,55]. These procedures are usually successful but highly simplified. A wide range of empirical factors is required, for which knowledge is limited only to certain types of soil. One of the major simplifications of these methods is the assumption that the bond stress at the soil–grout interface is mobilized uniformly along the whole fixed length. The result of these methods is usually only the ultimate capacity, with no additional information about the displacement required to mobilize this ultimate force. As outlined above, most of the scientific research on anchors involves a pull-out test. These are not common studies, but they are well documented in the literature. Acceptance tests are standardized and performed almost on a mass scale, but anchor displacement and creep velocity are only assessed in terms of compliance with standard values. Acceptance tests are of underestimated value, because analysis of displacements over time enables spatial evaluation of the rheological parameters of the soil.

In view of the numerous factors that affect the load capacity of anchors, the performance of acceptance tests for temporary structures is justified. The use of the full range of anchor acceptance test results is innovative; it allows the assessment of the variability of anthropogenic soil, which is not fully revealed by a typical discrete borehole investigation.

## 7. Conclusions

The authors focused on presenting an indirect research method to describe the heterogeneity of embankments. Based on the anchor creep model, the authors identified the heterogeneity of the material, the lack of spatial correlation between its properties and the large dispersion of the (rheological) parameters γ_1_, γ_2_ and θ_2_. The variability of the substrate–anchor system was investigated indirectly. The directly obtained experimental results allow the anchors to be accepted as a temporary support element for a retaining wall, according to the standard.

Geostatistical methods were used to investigate the variability of the mechanical features of the subsoil in the cross-section of the south and north wings of the tunnel wall protection. The values of anthropogenic embankment features were determined to have high variability and heterogeneity. The high variability prevented us from describing the rheological features of homogenized subsoil material and anchors by one theoretical semivariogram model. Linear and spherical models were used, and in each case, the effect of nuggets describing the randomness of the phenomenon plays an important role. Even at small distances between anchors, a significant value of semivariance—differentiation of mechanical properties—can be observed. This confirms the thesis of the high variability of parameters and, indirectly, of substrate characteristics. An important conclusion from the presented analysis is the confirmation of the necessity of acceptance testing for each anchor in the studied area. The presented method allows us to reduce the number of necessary anchor tests in the case of more homogeneous soils. In place of in situ testing, numerical simulation (randomization of features based on a semivariogram) can be performed, and the probability of failure can be investigated computationally. Analysis of the rheological model does not support the claim that the anthropogenic and fluvioglacial layers distinguished in the geotechnical documentation are homogeneous. The results obtained indicate major material heterogeneity. The spatial variability of the rheological model parameters means that the thickness of heterogeneous anthropogenic soils is higher than initially assumed; soils of postglacial origin have been relocated to and deposited in their current location. The notion of an uncontrolled embankment should be broadened to encompass the body of the heap as well. The presented research procedure and analysis of results also confirm the suitability of the designer’s (co-author of the article) decision to secure the temporary retaining wall with anchors. By adjusting the tension force and subjecting each anchor to an acceptance test, it was possible to achieve partial independence of the significant variability of soil characteristics. The spatial variability of the creep model parameters can be considered a new element in the process of determining the geodiversity of the subsoil. Given the variability of the mechanical features of the anthropogenic subsoil, the description of the geotechnical situation illustrates, first of all, the activities of humans and their omissions in the erection of embankments. It is unlikely that we will be able to discover the detailed reasons for this heterogeneity.

## Figures and Tables

**Table 1:** Model parameters with their direct statistics.

**Parameter**	**South Wing**		
	**Mean**	**Median**	**SD**
$\gamma_{1}$[MN/m]	9.625	9.311	2.420
$\gamma_{2}$[MN/m]	8.42 × 10^2^	5.16 × 10^2^	8.95 × 10^2^
$\theta_{2}$[m/s /MN]	3.83 × 10^3^	3.25 × 10^3^	2.77 × 10^2^
	**North Wing**		
	**Mean**	**Median**	**SD**
$\gamma_{1}$[MN/m]	9.863	9.917	1.440
$\gamma_{2}$[MN/m]	9.66 × 10^2^	7.97 × 10^2^	6.81 × 10^2^
$\theta_{2}$[m/s /MN]	4.83 × 10^3^	3.90 × 10^3^	4.61 × 10^2^
	**Both Wings**		
	**Mean**	**Median**	**SD**
$\gamma_{1}$[MN/m]	9.744	9.447	1.980
$\gamma_{2}$[MN/m]	9.04 × 10^2^	7.34 × 10^2^	7.92 × 10
$\theta_{2}$[m/s /MN]	4.330 × 10^3^	3.480 × 10^3^	3.81 × 10^2^

**Table 2:** The correlation matrix between model parameters for all results.

Parameter (from Each Test)	Correlation between Parameters
$\gamma_{1},\gamma_{2}$	0.2586
$\gamma_{2},\theta_{2}$	0.8481
$\gamma_{1},\theta_{2}$	0.3271

**Table 3:** Parameters of theoretical semivariogram models for the south wing.

Parameter	Model	Nugget	Sill	Range
$\gamma_{1}$	Linear	5.224	2.816	30.984
$\gamma_{2}$	Nugget	0.777 × 10^6^	-	-
$\theta_{2}$	Nugget	7.091 × 10^6^	-	-

**Table 4:** Parameters of theoretical semivariogram models for the north wing.

Parameter	Model	Nugget	Sill	Range
$\gamma_{1}$	spherical	0.392	1.077	5.879
$\gamma_{2}$	spherical	0.000	0.350 × 10^6^	6.170
$\theta_{2}$	spherical	0.000	14.884 × 10^6^	6.898

## Data Availability

https://drive.google.com/file/d/1S9YLbNIIwranZonHLkcbNmQX5ps5c62V/view?usp=sharing, accessed on 30 June 2021.

## References

[1] Baca M., Muszyński Z., Rybak J., Żyrek T. (2016). The application of geodetic methods for displacement control in the self-balanced pile capacity testing instrument. Advances and Trends in Engineering Sciences and Technologies. Proceedings of the Advances and Trends in Engineering Sciences and Technologies, Tatranská Štrba, Slovakia, 27–29 May 2015.

[2] Wujanz D., Neitzel F., Hebel H.P., Linke J., Busch W. (2013). Terrestrial radar and laser scanning for deformation monitoring: First steps towards assisted radar scanning. ISPRS Ann. Photogramm. Remote Sens. Spat. Inf. Sci..

[3] Oats R.C., Escobar-Wolf R., Oommen T. (2017). A Novel Application of Photogrammetry for Retaining Wall Assessment. Infrastructures.

[4] Górska K., Muszyński Z., Rybak J. (2013). Displacement monitoring and sensitivity analysis in the observational method. Stud. Geotech. Mech..

[5] Oskouie P., Becerik-Gerber B., Soibelman L. (2016). Automated measurement of highway retaining wall displacements using terrestrial laser scanners. Autom. Constr..

[6] Borana L., Yin J.-H., Singh D.N., Shukla S.K., Pei H.F. (2017). Influences of Initial Water Content and Roughness on Skin Friction of Piles Using FBG Technique. Int. J. Géoméch..

[7] Phoon K.-K., Quek S.T., An P. (2003). Identification of Statistically Homogeneous Soil Layers Using Modified Bartlett Statistics. J. Geotech. Geoenvironmental Eng..

[8] Sujkowski Z., Różycki S. (1937). Geology of Warsaw, Wyd.Wod. i Kan. oraz Wydz. Techn. Zarządu Miejskiego M.St. Warszawy, Warszawa, Poland. http://mbc.cyfrowemazowsze.pl/dlibra/doccontent?id=6367.

[9] Majerowicz A., Skoczylas J., Wojcik A. (1999). Petro Archaeological research in Lower Silesia. Przegląd Geol..

[10] Dobrzycki P., Ivannikov A., Rybak J., Shkodkina V.O., Tyulyaeva Y. (2019). The impact of Rapid Impulse Compaction (RIC) of large non-cohesive material deposits on the surrounding area. IOP Conf. Ser. Earth Environ. Sci..

[11] Zástĕrová P., Niemiec D., Marschalko M., Durd’Ák J., Duraj M., Yilmaz I., Drusa M. (2016). Analysis the Purposes of Land Use Planning on the Hard Coal Tailing Dumps. IOP Conf. Ser. Earth Environ. Sci..

[12] Machowski R., Rzętała M.A., Rzętała M., Solarski M. (2016). Geomorphological and hydrological effects of subsidence and land use change in industrial and urban areas. Land Degrad. Dev..

[13] PN-EN ISO 14688-1 (2017). Geotechnical Identification and Testing—Identification and Classification of Soil—Part 1: Identification and Description.

[14] Rybak J., Gorbatyuk S.M., Bujanovna-Syuryun K.C., Khairutdinov A.M., Tyulyaeva Y.S., Makarov P.S. (2021). Utilization of Mineral Waste: A Method for Expanding the Mineral Resource Base of a Mining and Smelting Company. Metallurgist.

[15] Drusa M. (2015). Numerical Verification of Geotechnical Structure in Unfavourable Geological Conditions—Case Study. Geosci. Eng..

[16] Batog A., Stilger-Szydło E. (2018). Low-Strength Substrates and Anthropogenic Soils in Transportation Engineering. Stud. Geotech. Mech..

[17] Rybak J., Kongar-Syuryun C., Tyulyaeva Y., Khayrutdinov A. (2021). Creation of Backfill Materials Based on Industrial Waste. Minerals.

[18] Ekici A., Huvaj N. A review on the use of marginal fills for geogrid-reinforced walls and slopes. Proceedings of the 6th European Geosynthetics Congress.

[19] Sobótka M., Łydżba D. (2019). Live load effect in soil-steel flexible culvert: Role of apparent cohesion of backfill. Eur. J. Environ. Civ. Eng..

[20] Bagińska I., Kawa M., Janecki W., Hicks M.A., Pisanò F., Peuchen J. (2018). Estimation of spatial variability properties of mine waste dump using CPTu results—Case study. Cone Penetration Testing 2018, Proceedings of the 4th International Symposium on Cone Penetration Testing (CPT’18), Delft, The Netherlands, 21–23 June 2018.

[21] Gabryś K., Soból E., Sas W. (2021). Physical, Deformation, and Stiffness Properties of Recycled Concrete Aggregate. Sustainability.

[22] Batog A., Stilger-Szydło E. (2018). Stability of Road Earth Structures in the Complex and Complicated Ground Conditions. Stud. Geotech. Mech..

[23] Năpăruş-Aljančič M., Pătru-Stupariu I., Stupariu M.-S. (2017). Multiscale wavelet-based analysis to detect hidden geodiversity. Prog. Phys. Geogr. Earth Environ..

[24] Reynard E., Brilha J. (2017). Geoheritage: Assessment, Protection, and Management.

[25] Bogdański J., Wróblewski A., Biernacki Z., Kazimierski J. (1990). Geomorphological Conditions. The Natural Environment of Warsaw.

[26] Kamińska M., Skrok Z. (1986). Warsaw—Warsaw Citadel. Port Kaponiera. Inf. Archeol..

[27] Fallavollita F., Ugolini A. (2017). New methodologies for the documentation of fortified architecture in the state of ruins. Int. Arch. Photogramm. Remote. Sens. Spat. Inf. Sci..

[28] Sabatini P.J., Pass D.G., Bachus R.C. (1999). Ground anchors and anchored systems. Geotechnical Engineering Circular.

[29] Gorska K., Wyjadłowski M. (2012). Analysis of displacement of excavation based on inclinometer measurements. Stud. Geotech. Mech..

[30] Biernacki Z., Teysseyre-Sierpińska M., Pietrusiewicz W. (2000). Map, Vistula River Range with Warsaw Slope. Geomorphology.

[31] Pawlak J., Teysseyre-Sierpińska M. (2006). Ecophysiographic Survey to Study the Conditions and Directions of Spatial Development In the Capital City of Warsaw.

[32] Caban W. (2018). The Nineteenth-Century Ideas of Polish Roads to Independence. Rocz. Inst. Eur. Srod.-Wschod..

[33] Yamshanov I., Goryunov V., Murgul V., Vatin N. (2014). Neogothic Public and Industrial Buildings in the Russian Empire XIX Century. Adv. Mater. Res..

[34] Śledź I. (2016). The Warsaw Citadel in the Years 1830–1864 Spatial Changes in Żoliborz in the Light of Archival Sources.

[35] Zieleniewska-Kasprzycka M., Borkowski W., Zieleniewska-Kasprzycka M. (2018). Results of archeological survey in the area of the former Protestant cemetery in the north-east section of the Warsaw Citadel. The Unknown History of the Cemetery from the Warsaw Citadel.

[36] Langins J. (2004). Conserving the Enlightenment: French Military Engineering from Vauban to the Revolution. Massachusetts Institute of Technology.

[37] Marten B., Meyer N. (2012). Festungsbau. Geometrie—Technologie-Sublimierung.

[38] Taras A.E. (2018). Anatomy hatred, Russian-Polish Conflicts in XVIII-XX th Century.

[39] Białkiewicz J. (2017). Katyń Museum in the Warsaw Citadel—Historic object in the interpretation of Modern Museum Architecture. J. Herit. Conserv..

[40] Geoteko Ltd (2013). Construction of the Complex along with Technical Infrastructure. Geotechnical Foundation Conditions.

[41] Xu H., Lu H., Qian Q. (2002). Creep Damage Effects of Pulling Grouting Anchor in Soil. Chin. J. Geotech. Eng..

[42] Xu H.-F., Wang F.-J., Cheng X.-X. (2007). Pullout creep properties of grouted soil anchors. J. Central South Univ. Technol..

[43] EN 1997-1 (2009). Eurocode 7: Geotechnical Design—Part 1: General Rules.

[44] EN 1537 European Standard (2013). Execution of Special Geotechnical Work—Ground Anchors.

[45] Burland J., Brown M., Skinner H.D., Chapman T. (2012). ICE Manual of Geotechnical Engineering. ICE Manual of Geotechnical Engineering.

[46] EN ISO 22477-5 (2018). Geotechnical Investigation and Testing—Testing of Geotechnical Structures Part 5: Testing of Grouted Anchors.

[47] Štefaňák J., Mica L., Chalmovský J., Leiter A., Tichý P. (2017). Full-scale Testing of Ground Anchors in Neogene Clay. Procedia Eng..

[48] Tomanovic Z. (2014). Testing of creep phenomena on soft rock. Gradjevinski Mater. I Konstr..

[49] Tomanovic Z., Miladinovic B., Zivaljevic S. (2015). Criteria for defining the required duration of a creep test. Can. Geotech. J..

[50] Bhat D.R., Bhandary N.P., Yatabe R. (2013). Residual-state creep behavior of typical clayey soils. Nat. Hazards.

[51] Dey A., Basudhar P.K. (2010). Applicability of Burger Model in Predicting the Response of Viscoelastic Soil Beds. GeoFlorida 2010.

[52] Findley W.N., Lai J.S., Onaran K., Christensen R.M. (1977). Creep and Relaxation of Nonlinear Viscoelastic Materials with an Introduction to Linear Viscoelasticity. J. Appl. Mech..

[53] Matheron G. (1963). Principles of geostatistics. Econ. Geol..

[54] Littlejohn G.S. (1980). Design estimation of the ultimate load-holding capacity of ground anchors. Ground Eng..

[55] Ostermeyer H. Construction, carrying behavior and creep characteristics of ground anchors. Proceedings of the Conference on Diaphragm Walls and Anchorages.

